# General health versus tumor stage: determinants of survival in Merkel cell carcinoma assessed by sentinel lymph node biopsy

**DOI:** 10.1007/s00432-026-06485-x

**Published:** 2026-04-28

**Authors:** Thilo Gambichler, Ekaterina Heinzer, Nessr Abu Rached, Hans-Joachim Schulze, Kirsten Noah, Silke C. Hofmann, Ulrich Wesselmann, Ralf Gutzmer, Stefanie Boms, Laura Susok, Sera S. Weyer-Fahlbusch, Alexander Kreuter, Julia Hyun, Valentina L. Müller, Rosanna Auer, Jürgen C. Becker

**Affiliations:** 1 Department of Dermatology, Dortmund Hospital gGmbH, University of Witten/Herdecke, Dortmund, Germany; 2 Department of Dermatology, Christian Hospital Unna, Unna, Germany; 3 Department of Dermatology, Skin Cancer Center, Ruhr-University Bochum, Bochum, Germany; 4 Department of Dermatology and Dermato-Histo-Pathology, Skin Cancer Center, Fachklinik Hornheide, Münster, Germany; 5 Center for Dermatology, Allergology and Dermatosurgery, Helios University Hospital Wuppertal, University of Witten/Herdecke, Wuppertal, Germany; 6 Department of Dermatology, Johannes Wesling Medical Center Minden, Ruhr University Bochum, Minden, Germany; 7 Department of Dermatology, Venereology and Allergology, University of Witten/Herdecke, HELIOS St. Elisabeth Hospital Oberhausen, Oberhausen, Germany; 8 Department of Dermatology, Venereology, and Allergology, Helios St. Johannes Hospital Duisburg, University of Witten/Herdecke, Duisburg, Germany; 9 Departments of Translational Skin Cancer Research and Dermatology, University Hospital Essen, Essen, Germany; 10 German Cancer Consortium (DKTK), Partner Site Essen/Düsseldorf and German Cancer Research Center (DKFZ), Heidelberg, Germany

**Keywords:** Cancer, Performance status, Elderly, Prognosis

## Abstract

**Purpose:**

Overall survival (OS) of Merkel cell carcinoma (MCC) patients is strongly influenced by health. Sentinel lymph node biopsy (SLNB) is recommended for staging. We evaluated whether SLNB is associated with OS in clinically node-negative MCC and contrasted tumor factors with patient frailty.

**Methods:**

STROBE-compliant cohort across eight centers in Germany (2004–2024). We included 271 primary stage I–II MCC; 167 underwent SLNB and 104 did not. The primary outcome was OS; disease-specific survival (DSS) and progression-free probability (PFP) were secondary. Kaplan–Meier and Cox models were used. Confounding by indication was addressed with 1:1 propensity score matching and sensitivity analyses.

**Results:**

Patients receiving SLNB were younger (median 74 vs 82 years;* p* < 0.001) and less comorbid (Charlson 4 vs 5). Ten-year OS was 69.5% with SLNB versus 45.2% without (log-rank* p* < 0.0001); unadjusted HR 0.34 (95% CI 0.20–0.59). In the matched cohort, SLNB remained associated with lower all-cause mortality (HR 0.56, 95% CI 0.34–0.93;* p* = 0.024). DSS did not differ (HR 1.09, 95% CI 0.55–2.13; * p*= 0.81). For PFP, unadjusted curves favored SLNB (*p*=0.0045), but the matched analysis was not significant (HR 0.53, 95% CI 0.23–1.26). Sensitivity analyses suggested benefit: overlap weighting HR 0.49 (95% CI 0.33–0.73;* p* = 0.00045) and a stage-restricted match HR 0.36 (95% CI 0.13–0.99; *p*= 0.048).

**Conclusions:**

SLNB was associated with improved OS after adjustment, supporting its role in staging and risk stratification. The absence of DSS and matched PFP differences highlights the influence of overall health; residual confounding by indication cannot be excluded.

**Supplementary Information:**

The online version contains supplementary material available at 10.1007/s00432-026-06485-x.

## Introduction

Merkel cell carcinoma (MCC) is a highly aggressive skin cancer with neuroendocrine differentiation. Its incidence among Whites living in the Northern hemisphere is approximately 0.7 per 100,000 person‑years and has risen steadily over recent decades (Lugowska et al. 2024; Lodde et al. 2025; Becker et al. 2023). MCC shows a marked propensity for regional and distant spread: up to one‑third of clinically node‑negative patients harbour occult nodal metastases at diagnosis (Becker et al. 2023; Shafique et al. 2025). Nodal status is the strongest predictor of recurrence and survival in primary MCC (Lugowska et al. 2024; American Joint Committee on Cancer 2017). Accordingly, sentinel lymph node biopsy (SLNB) is recommended to improve staging accuracy in clinically node‑negative disease (Wong et al. 2012). Early single‑centre and population‑based studies indicated that SLNB positivity identifies a subgroup at markedly higher risk for recurrence and disease‑specific mortality (Su et al. 2002; Ko et al. 2016; Conic et al. 2019). Meta‑analyses have reinforced the prognostic significance of sentinel node status, reporting pooled SLN positivity rates of 20–40% with worse outcomes among SLN‑positive patients (Sadeghi et al. 2014; Borgognoni et al. 2024). Nevertheless, systematic reviews in non‑melanoma skin cancers highlight underutilization of SLNB and inconsistent adherence to guideline recommendations (Borgognoni et al. 2024; Lewis et al. 2023). Most prior reports did not directly compare cohorts treated with SLNB to those in whom SLNB was omitted, limiting inference about whether the decision to perform SLNB itself is associated with long‑term outcomes. While SLNB is primarily a staging procedure that can guide adjuvant treatment, it could theoretically confer a direct prognostic benefit by removing micrometastatic disease or altering immune surveillance (Kanakopoulos et al. 2024; Lamb et al. 2018; Nan Tie and Kok 2022). However, any apparent survival advantage among patients undergoing SLNB may reflect selection bias: the decision to perform SLNB often favours younger patients with better performance status and fewer comorbidities. Disentangling a direct therapeutic effect from confounding by patient selection is therefore challenging in retrospective cohorts. To address this question, we conducted a retrospective multicentre analysis, reported in accordance with STROBE, explicitly incorporating tumor‑centric factors and available patient‑centric proxies (age and Charlson Comorbidity Index [CCI]) to compare survival and recurrence outcomes between patients who did and did not undergo SLNB (Scampa et al. 2023; Vordermark and Höller 2023; Alexander et al. 2024; Palencia et al. 2021; John 2022; Perez et al. 2019). By transparently acknowledging undocumented selection factors and focusing on measurable health indicators, we aim to inform a more differentiated, patient‑centred understanding of the role of SLNB in MCC.

## Methods

### Patients

The study adhered to the STROBE guidelines (Table S1). We performed a retrospective multicentre cohort study across eight skin cancer centres in North Rhine‑Westphalia, Germany (Bochum, Dortmund, Duisburg, Minden, Oberhausen, Hornheide [Münster], Unna, Wuppertal) covering the years 2004–2024. The protocol was approved by the institutional ethics review board (#16 5985), and the study was conducted in accordance with the Declaration of Helsinki. We included 271 consecutive patients with a primary diagnosis of MCC and an indication for SLNB per national guidelines (clinical stage I–II). Baseline staging comprised lymph‑node ultrasound, thoraco‑abdominal computed tomography, and cranial magnetic resonance imaging. SLNB was recommended for all patients in the absence of formal contraindications; however, 104 patients did not undergo SLNB, likely due to frailty, comorbidities, restricted life expectancy, or patient refusal. These reasons were not consistently recorded; consequently, beyond age and CCI we lacked comprehensive data to adjust for all patient‑ or physician‑driven selection factors. Clinical work‑up, treatment, and follow‑up were performed according to national guidelines (Becker et al. 2023).

### Data collection and baseline variables

Key baseline covariates are summarized in Table 1. Age and sex were recorded at first presentation. Tumor stage was assigned per AJCC 8th edition immediately after SLNB for SLNB patients and at initial diagnosis for non‑SLNB patients. Comorbidities, including immunosuppression and other immunodeficiencies, were quantified using the CCI. Variables and survival outcomes were extracted by a dermatologist (E.H.) using each centre’s electronic health records, a predefined structured data collection form, and a data dictionary developed with a senior consultant dermatologist (T.G.).

**Table 1:** Showing univariable analyses of baseline characteristics and outcome of Merkel cell carcinoma (MCC). patients with (n = 167) or without (n = 104) sentinel lymph node biopsy (SLNB)

Parameters	With SLNB	Without SLNB	*P*-value
*Age*			
Median (range) years	74 (35–92)	82 (58–100)	< 0.0001
*Sex*			
F/M	76/91 (45.5%/54.5%)	59/45 (56.7/43.3%)	= 0.09
*Charlson comorbidity index*			
median (range) years	4 (0–11)	5 (2–10)	= 0.0009
MCC localization**			
Head/neck	50 (29.9%)	61 (58.7%)	< 0.0001
Upper extremity	69 (41.3%)	22 (21.2%)	= 0.0024
Lower extremity	44 (26.3%)	16 (15.4%)	= 0.14
Trunk	4 (2.5%)	5 (4.8%)	= 1.0
Tumor stage at baseline*/^**^			
Stage I	75 (44.9%)	58 (55.8%)	n.s
Stage IIa	41 (24.6%	33 (31.7%)	n.s
Stage IIb	7 (4.2%)	13 (12.5%)	n.s
*Upstaged by SLNB*			
Stage IIIA	43 (25.7%)	N/A	N/A
*CLND for stage IIIA*			
No/yes	146/18 (89.2/10.8%)	N/A	N/A
*Adjuvant radiotherapy*			
No/yes	46/121 (27.5%/72.5%)	62/42 (59.6%/40.4%)	< 0.0001
*Disease relapse*			
No/yes	129/38 (77.2%/22.8%)	86/18 (82.7%/17.3%)	= 0.61
Median (IQR) follow-up for PFP (months)	47.3 [27.5–79.4]	21.5 [6.5–71.0]	< 0.0020
MCC relapse sites**			
Local, regional lymph nodes	15 (39.5%)	7 (38.9%)	= 1.0
Distant metastases or unknown	23 (60.5)	11 (61.1%)	
*CLND after first relapse*			
No/yes	153/14 (91.6.2/8.4%)	98/6 (94.2/5.8%)	= 0.57
Immunotherapy after relapse***			
No/yes	133/34 (79.6%/20.4%)	90/14 (86.4/13.6%)	0.20
Chemotherapy after relapse***			
No/yes	159/8	103/1	= 0.087
*DSS*			
MCC-death (no/yes)	141/26 (84.4%/15.6%)	91/13 (86.4%/13.6%)	= 0.48
Median (IQR) follow-up for DSS (months)	43.5 (16.9–62.7)	12.1 (4.3–36)	< 0.0001
*OS*			
Any death (no/yes)	131/36	79/25	= 0.63
Median (IQR) follow-up for OS (months)	43.5 (19.1–68)	19.2 (4.3–36)	= 0.0008

^*^ immediately after SLNB or at first diagnosis without SLNB (AJCC 8th edition); ** Bonferroni corrected for several groups; CLND = completion lymph node dissection (after positive SLNB or after first relapse); IQR, interquartile range; DSD = disease-specific survival; OS = overall survival; PFP = progression-free probability. ***unresectable metastatic disease

### SLNB procedure

SLNB was performed predominantly under general anaesthesia. Lymphatic mapping was conducted by intradermal injection of technetium‑99m sulfur colloid adjacent to the tumor or biopsy site, followed by gamma imaging to identify draining basins. In select cases, intradermal blue dye was injected intraoperatively. Nodes appearing blue and/or exhibiting radioactivity greater than 10% of the ex vivo counts were excised as sentinel lymph nodes, as described by (Wong et al. 2012)) Pathologic examination followed the recommendations by Su et al. (2002)) Immunohistochemical stains typically included cytokeratin‑20 (CK20) and/or pan‑cytokeratin (Becker et al. 2023).

### Outcomes

The primary outcome was overall survival (OS), defined from diagnosis to death from any cause; survivors were censored at last known contact. Disease‑specific survival (DSS) was defined as time from diagnosis to death due to MCC; deaths from other causes were treated as competing events, and survivors were censored at last follow‑up. Progression‑free probability (PFP) was defined as the Kaplan–Meier‑estimated probability of remaining free from the composite event (first MCC recurrence or death from any cause) up to time t and was reported at prespecified time points (e.g., 12, 24, and 60 months). For modelling and hypothesis testing, we analysed the corresponding time‑to‑event (time to first recurrence or death); deaths were counted as events.

### Statistics

Analyses were performed using MedCalc Software v22.014 (Ostend, Belgium) and R/RStudio (version 4.4.3; packages prodlim, survival, and optmatch). Sample size was determined a priori via Schoenfeld’s formula: assuming two‑sided α = 0.05, 80% power to detect HR = 2.0 for OS, equal SLNB/non‑SLNB allocation, and a 25% anticipated OS event rate, a minimum of 261 patients was required; our cohort of 271 thus afforded adequate power. Missing data for key outcomes led to case‑wise exclusion. Data distribution was assessed with the D’Agostino–Pearson test; non‑normal variables are reported as medians with ranges or interquartile ranges. Survival curves were estimated by Kaplan–Meier and compared by log‑rank test. Follow‑up time for OS, DSS, and the time‑to‑composite event underlying PFP was estimated by the reverse Kaplan–Meier method, with median follow‑up defined as the time point at which the reverse KM survival curve fell to 50%. Multivariable Cox proportional hazards models included age (continuous, HR per 10‑year increase), sex (male vs female), clinical stage (I vs II), CCI (continuous, HR per one‑point increase), SLNB performance (no vs yes), tumor localization, and adjuvant radiotherapy. The proportional hazards assumption was verified via Schoenfeld residuals and held for all covariates; ties were handled by the Breslow approximation (MedCalc default). For DSS, non‑MCC deaths were treated as competing events in a cause‑specific Cox model. To minimize residual confounding between SLNB and non‑SLNB groups, we performed propensity‑score matching (PSM) by estimating each patient’s propensity to undergo SLNB via logistic regression on age, sex, CCI, clinical AJCC stage (I vs II), tumor localization, and adjuvant radiotherapy. We then performed 1:1 nearest‑neighbour matching with a caliper of 0.05 on the propensity score. Matched pairs (85 SLNB vs 85 non‑SLNB) were used for univariable Cox regression analyses of OS, DSS, and the time‑to‑composite event; PFP was reported at fixed times. Covariate balance was assessed using absolute standardized mean differences, with values < 0.20 considered acceptable. In addition, robustness of the results was evaluated by varying caliper widths, restricting analyses to common support (stage-restricted matching), and applying overlap weighting to approximate the average treatment effect in the overlap population.

## Results

### Baseline characteristics

**Fig. 1:**
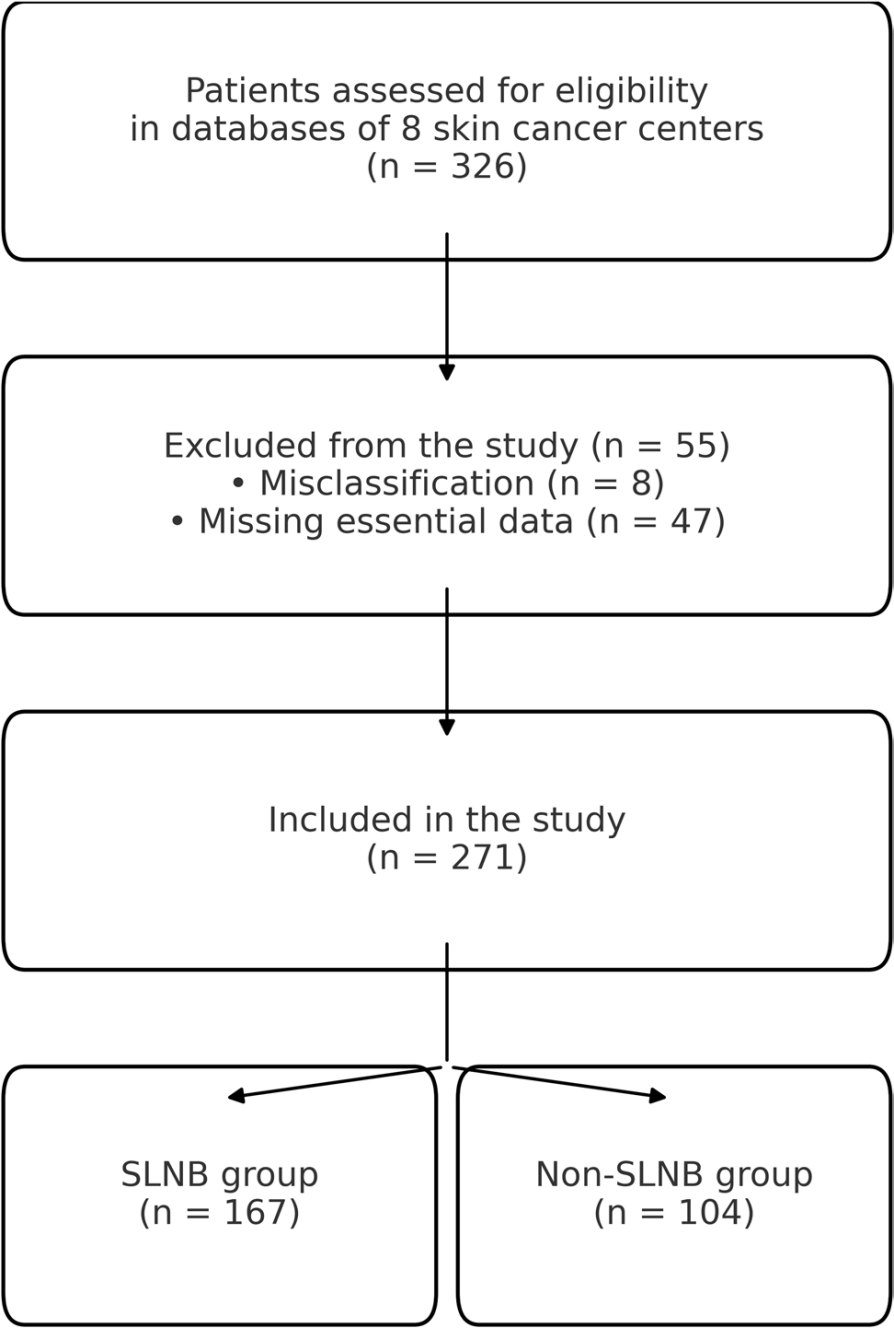
Flow diagram of patient inclusion. Of 326 Merkel cell carcinoma patients screened across eight tertiary skin cancer centers (2004–2024), 55 were excluded due to missing data [survival data 36/55 (65.5%) and CCI data 11/55 (20%)] or misclassification [8/55 (14.5%)], yielding 271 patients for analysis with 167 in the SLNB group and 104 in the non-SLNB group

As shown in the flow diagram (Fig. 1), 271 patients with primary MCC were included; 167 (61.6%) underwent SLNB at diagnosis and 104 (38.4%) did not. Of the initial 326 patients, 55 were excluded due to missing data [survival data 36/55 (65.5%) and CCI data 11/55 (20%)] or misclassification [8/55 (14.5%)]. SLNB patients were significantly younger (median 74 vs 82 years; *p* < 0.0001) and had a lower comorbidity burden (median CCI 4 vs 5; *p* = 0.0009). Although guidelines recommend adjuvant radiotherapy irrespective of SLNB status, radiotherapy was administered more often in the SLNB cohort (72.5% vs 40.4%; *p* < 0.0001).

### Relapse and mortality rates

Overall, 56/271 patients (20.7%) experienced a disease relapse; 38/167 (22.8%) in the SLNB group and 18/104 (17.3%) in the non‑SLNB group (χ^2^ = 1.16; *p* = 0.28).

### Survival analyses

**Fig. 2:**
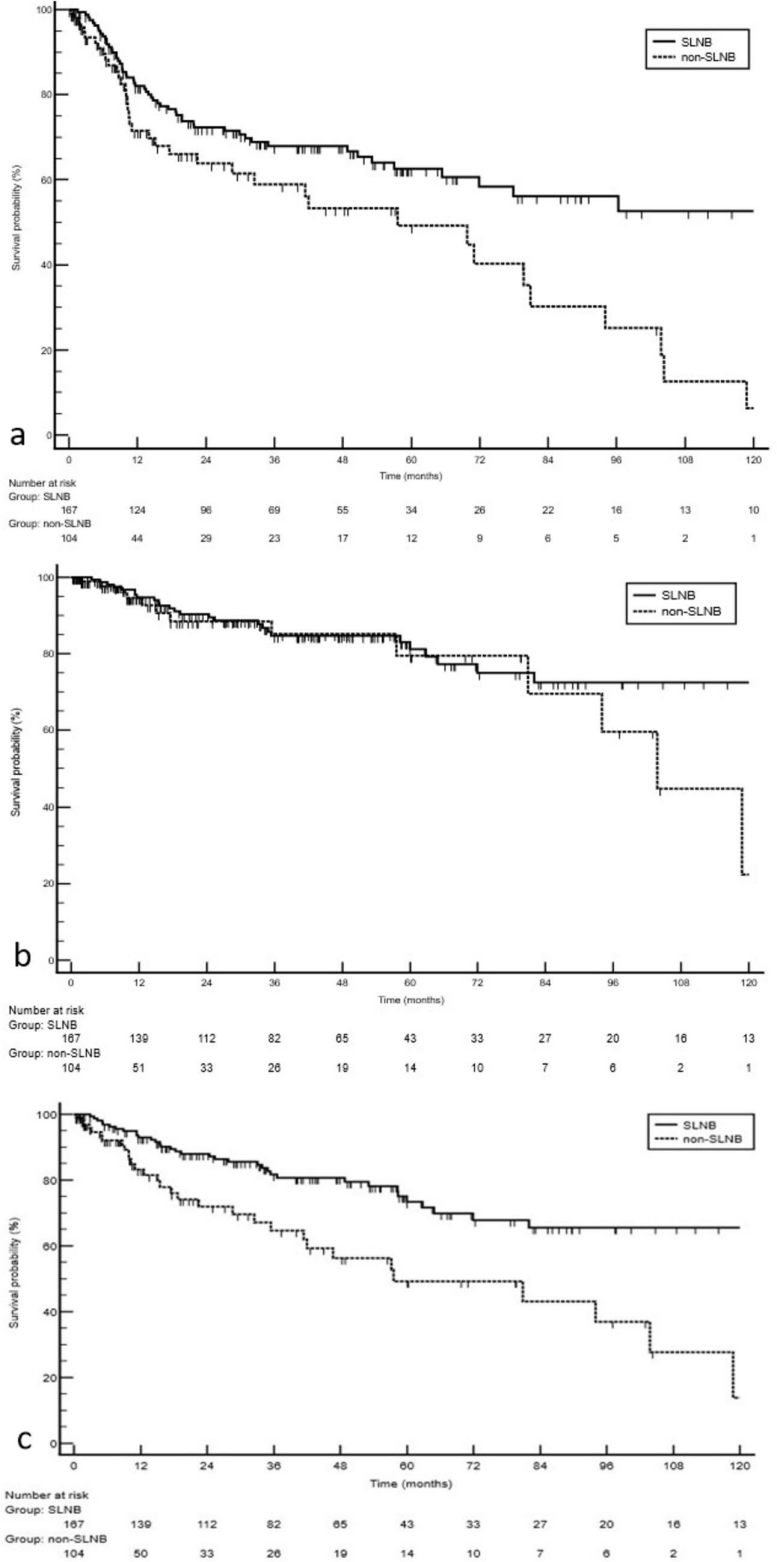
Progression-free probability (PFP), disease-specific survival (DSS) and overall survival (OS) in patients with Merkel cell carcinoma stratified by sentinel lymph node biopsy (SLNB) performance. (**a**) PFP (composite: recurrence or death). The median time to the composite event was not reached in the SLNB group, whereas it was 57.6 months (IQR 24.3–93.5) in the non-SLNB group; log-rank Z = –2.84,* p* = 0.0045; Cox HR (SLNB vs non-SLNB = 0.55 (95% CI 0.36–0.84). PFP at 12/24/60 months: 82.0%/72.3%/62.6% (SLNB) vs 71.5%/63.8%/49.2% (non-SLNB). (**b**) DSS. Median DSS was 35.4 months (IQR 16.9–62.7) in SLNB vs 10.9 months in non-SLNB; log-rank *p*= 0.26; HR = 0.66 (95% CI 0.32–1.36). (**c**) *OS*. Median OS was not reached in SLNB vs 57.6 months (95% CI 41.4–103.8) in non-SLNB; 10-year OS 69.5% vs 45.2%; log-rank χ^2^ = 35.56 (df = 1),* p* < 0.0001; adjusted HR = 0.34 (95% CI 0.20–0.59). Solid line = SLNB (n = 167), dashed line = non-SLNB (n = 104); tick marks indicate censored observations. Numbers at risk are shown below the x-axis

Median time to the composite endpoint (first recurrence or death) was not reached in the SLNB group, as more than half of patients remained event‑free at last follow‑up. Kaplan–Meier curves demonstrated superior PFP in the SLNB group compared with the non‑SLNB group (log‑rank Z = –2.84; *p* = 0.0045, Fig. 2). Corresponding PFP at 3, 5, and 10 years were 68.5% (95% CI 60.1–75.5), 61.5% (51.9–69.8), and 49.5% (36.9–60.9) in the SLNB cohort versus 53.5% (40.6–64.8), 39.2% (25.2–52.9), and not estimable in the non‑SLNB cohort (Table 2). DSS did not differ significantly between groups by time‑to‑event analysis (HR 0.66; 95% CI 0.32–1.36; *p* = 0.26). Median OS was not reached in the SLNB group versus 57.6 months (95% CI 41.4–103.8) in the non‑SLNB group; 10‑year OS rates were 69.5% vs 45.2% (log‑rank χ^2^ = 35.56; *p* < 0.0001; HR 0.34; 95% CI 0.20–0.59). Restricted mean survival time at 60 months was 51.8 months (95% CI 48.98–54.57) in the SLNB group vs 42.6 months (95% CI 37.20–48.05) in the non‑SLNB group, yielding an absolute gain of 9.2 months (95% CI 3.05–15.26; *p* = 0.0033). Among the 167 SLNB‑treated patients, 43 (25.7%) had a positive sentinel node, upstaging them to stage IIIA and identifying a high‑risk subgroup across survival endpoints. Stage IIIA had shorter OS compared to SLNB‑negative patients (log‑rank χ^2^ = 7.25; *p* = 0.0071; HR 3.04; 95% CI 1.35–6.81), with a median OS of 82.0 months (95% CI 34.9–82.0) versus not reached for pathologically confirmed stage I/II (pI/pII). DSS was also inferior in stage IIIA (log‑rank χ^2^ = 9.89; *p* = 0.0017; HR 4.56; 95% CI 1.77–11.73), with a median DSS of 82.0 months (95% CI 34.9–82.0) versus not reached in pI/pII. However, the shorter PFP in stage IIIA did not reach statistical significance (log‑rank χ^2^ = 1.42; *p* = 0.23; HR 1.61; 95% CI 0.74–3.54).

**Table 2:** Progression-free probability (PFP; composite: first recurrence or death) at 3, 5, and 10 years for patients with Merkel cell carcinoma (MCC) who underwent sentinel lymph node biopsy (SLNB) versus those without SLNB

Time (years)	SLNB (n = 167)	Non-SLNB (n = 104)
3	68.5% (95% CI 60.1–75.5)	53.5% (95% CI 40.6–64.8)
5	61.5% (95% CI 51.9–69.8)	39.2% (95% CI 25.2–52.9)
10	49.5% (95% CI 36.9–60.9)	n.e

PFP rates were derived from Kaplan–Meier analysis and are presented with 95% confidence intervals (CI). n.e. = not estimable due to < 10 patients at risk at the time point

### Multivariable Cox regression

We fitted multivariable Cox models for PFP, OS, and DSS incorporating age, sex, CCI, tumor localization and stage, SLNB performance, and adjuvant radiotherapy. Each full model significantly outperformed its null counterpart (PFP χ^2^ = 28.72; OS χ^2^ = 52.27; DSS χ^2^ = 34.45; all *p* < 0.003). For PFP, higher CCI and tumor stage II were independently associated with earlier progression or death (CCI HR = 1.18; 95% CI 1.07–1.29; stage II vs I HR = 1.99; 95% CI 1.07–3.68). Age, sex, localization, adjuvant radiotherapy, and SLNB performance were not significant for PFP. For DSS, stage II carried a 3.54‑fold increased hazard (95% CI 1.22–10.3; *p* = 0.020) and age a 1.09‑fold increase (95% CI 1.021–1.16; *p* = 0.0092). Comorbidity, SLNB, sex, localization, and adjuvant radiotherapy lacked independent significance for DSS. For OS, comorbidity remained a key predictor (CCI HR = 1.17 per point; 95% CI 1.03–1.33; *p* = 0.014) alongside stage II (HR = 2.60; 95% CI 1.31–5.60; *p* = 0.007) and age (HR = 1.06; 95% CI 1.01–1.10; *p* = 0.0095). SLNB was not independently associated with OS after adjustment. Harrell’s C‑indices indicated good discrimination (OS 0.74; DSS 0.74; PFP 0.67). No covariate violated the proportional‑hazards assumption (all Schoenfeld *p* > 0.10).

#### Propensity‑matched and weighted analyses

**Fig. 3:**
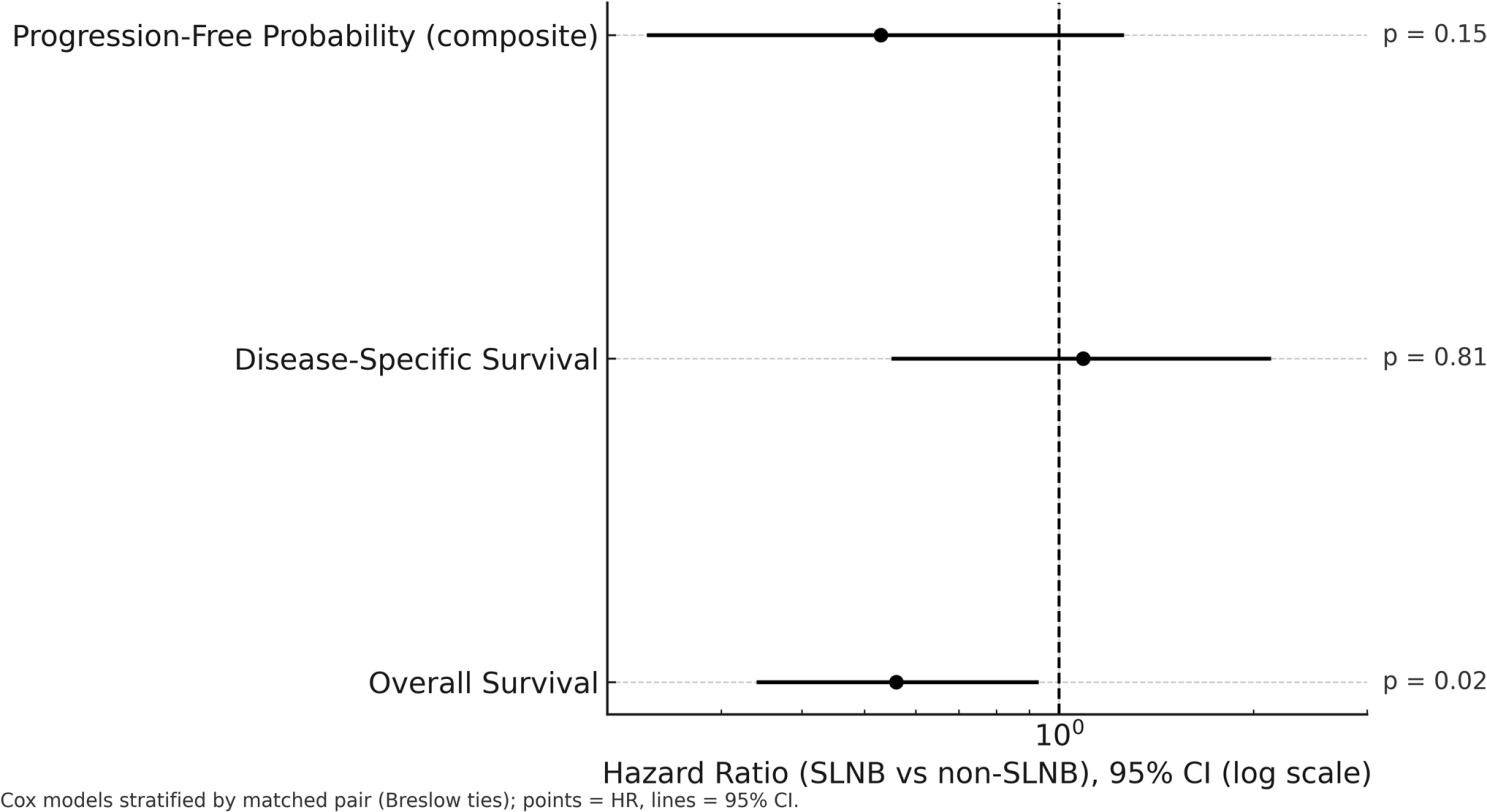
Forest plot of hazard ratios (HRs) comparing sentinel lymph node biopsy (SLNB) vs non-SLNB in the 1:1 propensity-score-matched cohort. Points show HRs on a log scale; horizontal lines indicate 95% confidence intervals; the vertical dashed line at HR = 1 denotes no effect. Progression-free probability (composite: recurrence or death): HR 0.53 (95% CI 0.23–1.26),* p* = 0.15. Disease-specific survival: HR 1.09 (95% CI 0.55–2.13),* p* = 0.81. Overall survival: HR 0.56 (95% CI 0.34–0.9,* p* = 0.02. All* p*-values two-sided; Cox models stratified by matched pair (Breslow ties)

PSM yielded 85 well‑balanced pairs (absolute standardized differences < 0.20). In the matched cohort (Table 3, Fig. 3), SLNB was associated with lower all‑cause mortality (OS HR = 0.56; 95% CI 0.34–0.93; *p* = 0.024), while DSS did not differ (HR = 1.09; 95% CI 0.55–2.13; *p* = 0.81). The time‑to‑composite event underlying PFP was not significantly different (HR non‑SLNB vs SLNB = 1.88; 95% CI 0.79–4.42; *p* = 0.15). Corresponding PFP values at 12/24/60 months in the matched set (caliper 0.05) were 74.8%/68.4%/60.5% (SLNB) vs 73.2%/63.2%/42.6% (non‑SLNB). Robustness checks confirmed the OS finding: with caliper 0.20 (matched N = 170) HR = 0.65 (95% CI 0.44–0.96; *p* = 0.03); with caliper 0.10 (matched N = 150) HR = 0.63 (95% CI 0.41–0.99; *p* = 0.04). In a stage I–II restricted match emphasizing common support (1:1, caliper 0.05; 52 pairs), pair‑stratified Cox showed a higher composite event hazard in non‑SLNB (HR = 2.80; 95% CI 1.01–7.77; *p* = 0.048). Overlap weighting achieved near‑perfect balance and indicated a lower composite event hazard with SLNB (HR = 0.49; 95% CI 0.33–0.73; *p* = 0.00045).

**Table 3:** Sensitivity analyses of the effect of sentinel lymph node biopsy (SLNB) on outcomes using 1:1 nearest-neighbour propensity-score matching on the logit of the propensity score with calipers of 0.20 and 0.10, a stage I–II restricted match, and an overlap-weighted analysis of the full cohort

Outcome	Analysis	M‑N	HR (95% CI)	*p* value
OS	1:1 nearest‑neighbour, caliper = 0.20 (logit PS)	170	0.65 (0.44–0.96)	0.03
OS	1:1 nearest‑neighbour, caliper = 0.10 (logit PS)	150	0.63 (0.41–0.99)	0.04
PFP (composite)	1:1 nearest‑neighbour, caliper = 0.20 (logit PS)	112	0.56 (0.25–1.27)	0.17
PFP (composite)	1:1 nearest‑neighbour, caliper = 0.10 (logit PS)	106	0.53 (0.23–1.26)	0.15
PFP (composite)	1:1 nearest‑neighbour, caliper = 0.05; stage I–II restricted	104	0.36 (0.13–0.99)	0.048
PFP (composite)	Overlap weighting (full cohort)	– †	0.49 (0.33–0.73)	0.00045

Propensity scores were estimated via logistic regression including age, sex, Charlson Comorbidity Index (CCI), clinical AJCC stage (I vs II), tumor site, and adjuvant radiotherapy. Matched N (M–N) denotes the total number of matched patients (2 × pairs). Hazard ratios (HR) and 95% confidence intervals (CI) were obtained from pair-stratified Cox proportional hazards models (Breslow ties); p values are two-sided. For OS, the outcome was time from diagnosis to death from any cause. For PFP (progression-free probability), rows report HRs for the time-to-composite event underlying the PFP curve, defined as time from first surgery to the earliest of first MCC recurrence or death from any cause. **HR < 1 favours SLNB**

SLNB, sentinel lymph node biopsy; PS, propensity score; HR, hazard ratio; CI, confidence interval; OS, overall survival; PFP, progression‑free probability; MCC, Merkel cell carcinoma; CCI, Charlson Comorbidity Index; AJCC, American Joint Committee on Cancer. †Overlap weighting uses the full cohort (N = 271); “M–N” (matched N) is not applicable

## Discussion

### Data and literature

This multicenter cohort of clinically node‑negative MCC provides several insights into the role of SLNB. Consistent with prior reports, performance of SLNB was associated with a marked improvement in unadjusted overall survival (10‑year OS 69.5% vs 45.2%) (Lodde et al. 2025; Shafique et al. 2025; Ko et al. 2016; Sadeghi et al. 2014; Delisle et al. 2022; Harounian et al. 2021; Sattler et al. 2013; Song et al. 2021). The survival advantage persisted, though attenuated, after propensity‑score matching. Interpreting this association requires caution. SLNB improves staging accuracy and facilitates risk‑adapted treatment, which may indirectly improve outcomes. Whether SLNB itself confers a direct therapeutic benefit by removing micrometastases remains unproven in MCC and has not been definitively established in melanoma.

Importantly, the observed dissociation between improved OS and unchanged DSS strongly suggests that non-cancer mortality plays a major role in this cohort. Patients selected for SLNB were younger and less comorbid, indicating that general health status rather than tumor biology likely drives a substantial proportion of the OS difference. In this context, SLNB appears to function as a proxy for patient fitness and treatment eligibility rather than a direct modifier of MCC-specific mortality. Competing risks, particularly age- and comorbidity-related mortality, are therefore critical when interpreting OS differences in MCC.

Our results are concordant with Delisle et al., who observed a 68% reduction in all‑cause mortality after SLNB (HR 0.32; 95% CI 0.23–0.45) in a propensity‑matched Canadian cohort, and with other series reporting improved OS without consistent DSS differences (Delisle et al. 2022; Harounian et al. 2021; Sattler et al. 2013; Song et al. 2021). In our data, DSS did not differ significantly between SLNB and non‑SLNB across analyses (HR 1.09; 95% CI 0.55–2.13; *p* = 0.81), mirroring several prior studies (Delisle et al. 2022; Smith et al. 2015). SLN positivity (stage IIIA) identified a high‑risk subgroup across OS and DSS endpoints in our cohort, in line with established literature. Nevertheless, adjuvant radiotherapy was more frequent in the SLNB group (72.5% vs 40.4%), which may reflect enhanced risk stratification after pathological staging or indicate that the same clinical factors precluding SLNB also influenced the decision to omit radiotherapy. Non‑SLNB patients were also older and had more head‑and‑neck primaries, features historically linked to poorer prognosis (Ma et al. 2023; Jacobs et al. 2021; Erstine et al. 2019; Jenkins et al. 2019). Although PSM and overlap weighting improved balance, residual confounding remains possible and may partly account for the observed OS benefit.

Overall, our data suggest that the principal advantage of SLNB is improved staging with downstream treatment selection rather than a demonstrated intrinsic effect on recurrence biology. Regarding covariate balance, propensity score matching achieved acceptable equilibrium across measured variables, with absolute standardized differences below 0.20. However, residual imbalance cannot be excluded, particularly for unmeasured confounders such as performance status, frailty, or physician decision-making. To address this, we complemented the primary matching approach with sensitivity analyses including different caliper widths, stage-restricted matching, and overlap weighting. Notably, the direction and magnitude of the association between SLNB and OS remained consistent across these specifications, supporting the robustness of the findings, although effect sizes were attenuated after adjustment. The composite PFP endpoint favoured SLNB in unadjusted analyses; in the primary matched analysis it was neutral overall but showed benefit in the stage‑restricted match and in the overlap‑weighted estimand. No adjustments for multiplicity were applied, and inferences should be regarded as exploratory. We prioritized estimation with effect sizes and uncertainty intervals over strict hypothesis testing. Recent data from a large retrospective cohort (n = 696) published by Dugan et al. (2026) this year further support an association between SLNB performance and improved oncologic outcomes, including RFS, DSS, and OS on multivariable analysis. Notably, patients in whom SLNB was omitted were older and more comorbid, consistent with our cohort. While these findings may suggest a broader survival benefit of SLNB, they likely also reflect differences in baseline patient fitness and treatment allocation. In contrast to this study, we did not observe a DSS advantage after adjustment, underscoring the potential impact of competing risks and residual confounding. Together, these data reinforce that SLNB is primarily a staging tool, while observed survival differences across studies should be interpreted in the context of patient selection (Dugan et al. 2026).

From a clinical perspective, these findings support a nuanced interpretation: SLNB should primarily be regarded as a staging and risk-stratification tool rather than a direct therapeutic intervention. In elderly or highly comorbid patients, omission of SLNB may be appropriate when procedural risks outweigh potential benefits, particularly if the results are unlikely to alter subsequent management. Accordingly, SLNB should be embedded within a shared decision-making framework that integrates tumor characteristics with patient fitness, comorbidity burden, and life expectancy. Kaplan–Meier curves were visually inspected for late separation and crossing hazards. Schoenfeld residual plots showed no meaningful departures from proportionality on visual review. Event adjudication relied on clinical documentation and imaging reports available in the electronic record. The analytic code and data dictionary are available on request to support reproducibility. The participating centers spanned urban and regional catchment areas, supporting external validity within our healthcare system. Future work should incorporate patient‑reported outcomes to complement survival‑based endpoints.

### Limitations and strengths

This study has several limitations. Its retrospective design introduces selection bias and precludes causal inference. MCPyV status was not uniformly assessed, limiting analyses by viral aetiology. Key confounders such as performance status, detailed comorbidity beyond the CCI, and socioeconomic factors were not captured. Follow‑up schedules varied across centres, potentially underestimating late recurrences. Documentation of the rationale for omitting SLNB was incomplete, limiting adjustment for selection factors. Nevertheless, the study has notable strengths. The relatively large, multicenter setting bolstered statistical power despite MCC’s rarity and enhanced generalizability. Data abstraction followed a centralized, standardized process with STROBE‑conformant reporting to ensure transparency and reproducibility. We prespecified a composite definition for PFP and complemented the primary matched analysis with overlap weighting and stage‑restricted sensitivity analyses to probe robustness. Model performance was summarized with Harrell’s C‑indices, and no proportional‑hazards violations were detected.

## Conclusions and clinical implications

In this multicenter cohort of clinical stage I–II MCC, SLNB was associated with substantially better unadjusted OS, and this association persisted after propensity-score matching, albeit attenuated. For PFP (composite) and DSS, the primary 1:1 matched analysis did not show statistically significant differences, although unadjusted PFP curves favoured SLNB and sensitivity analyses suggested a lower hazard of progression or death with SLNB. Multivariable models identified comorbidity (CCI), tumor stage II, and age as the principal determinants of outcome; SLNB itself was not independently prognostic after adjustment. The discordance between OS and DSS underscores the importance of competing risks and patient frailty in MCC and highlights that SLNB primarily reflects patient selection and staging rather than a direct therapeutic effect. Accordingly, SLNB should be interpreted primarily as a staging and risk-stratification tool. Its use should be individualized within a shared decision-making framework, particularly in elderly or comorbid patients, where the clinical benefit of the procedure may be limited. From a clinical and guideline perspective, our findings support current recommendations that advocate SLNB as a staging procedure in clinically node-negative MCC, while emphasizing individualized decision-making. In routine practice, the decision to perform SLNB should integrate tumor-related risk (e.g., tumor size, localization) with patient-related factors such as age, comorbidity burden, and life expectancy. In patients with substantial frailty or limited expected survival, the incremental value of SLNB for guiding treatment may be outweighed by procedural risks and patient burden. Conversely, in fit patients, SLNB provides critical prognostic information that may influence adjuvant treatment strategies and follow-up intensity. Accordingly, we propose that SLNB should be framed within a shared decision-making model, where its role as a staging and risk-stratification tool is clearly communicated, and expectations regarding survival benefit are contextualized in light of competing risks (Straker et al. 2022; Lambert et al. 2020).

## Supplementary Information

Below is the link to the electronic supplementary material.

Supplementary file1 (DOCX 32 KB)

## Data Availability

The data that support the findings of this study are available from the corresponding author upon reasonable request.
