# Supplementary material for: General health versus tumor stage: determinants of survival in Merkel cell carcinoma assessed by sentinel lymph node biopsy
Source: J Cancer Res Clin Oncol. 2026 Apr 28;152(4):100. doi: 10.1007/s00432-026-06485-x (PMC13125591; doi:10.1007/s00432-026-06485-x)
Supplement: Supplementary file 1 — Supplementary file1 (DOCX 32 KB) [file 432_2026_6485_MOESM1_ESM.docx]

# Completed STROBE Checklist

| Item No. | Recommendation | Addressed? | Location / Notes (page) |
| --- | --- | --- | --- |
| 1a | Indicate study design in title | Yes | Title page (p.1) |
| 1b | Informative, balanced summary in abstract | Yes | Abstract (p.2) |
| 2 | Background/rationale | Yes | Introduction (p.3) |
| 3 | Specific objectives, hypotheses | Yes | Introduction (p.4) |
| 4 | Key elements of study design early | Yes | Methods (p.5) |
| 5 | Setting, locations, dates of recruitment/exposure/follow-up | Yes | Methods (p.5) |
| 6a | Eligibility criteria, sources and selection methods; follow-up methods | Yes | Methods (p.6) |
| 6b | For matched studies, matching criteria and numbers | Yes | Methods – Propensity matching (p.6) |
| 7 | Definitions of outcomes, exposures, predictors, confounders | Yes | Methods – Data collection (p.7) |
| 8* | Data sources/measurement methods | Yes | Methods – Measurement (p.7) |
| 9 | Efforts to address bias | Yes | Methods – Statistical analysis (p.8) & Limitations (p.12) |
| 10 | Explanation of study size determination | Yes | Methods (p.8) |
| 11 | Handling of quantitative variables | Yes | Methods – Statistical analysis (p.9) |
| 12a | Statistical methods including confounding control | Yes | Methods – Statistical analysis (p.9) |
| 12b | Methods for subgroups/interactions | Yes | Methods – Statistical analysis (p.9) |
| 12c | How missing data were addressed | Yes | Methods – Statistical analysis (p.8, 10) |
| 12d | Cohort: handling loss to follow-up | Yes | Methods – Outcomes (p.10) |
| 12e | Sensitivity analyses | Partially | Methods – Sensitivity not performed (p.11) |
| 13* | Numbers at each stage | Yes | Results – Fig.1 (p.9) |
| 14* | Participant characteristics and missing data per variable | Yes | Results – Table 1 (p.10) |
| 14c | Summarize follow-up time | Yes | Results (p.10) |
| 15* | Numbers of outcome events over time | Yes | Results (p.10) |
| 16a | Unadjusted and adjusted estimates with precision | Yes | Results – Table 1 (p.10) |
| 16b | Report category boundaries | Yes | Methods – Statistical analysis (p.9) & Table 1 (p.10) |
| 16c | Translate relative risk into absolute risk | Yes | Results (p.10) |
| 17 | Other analyses (subgroup, interaction, sensitivity) | Partially | Results (p.11) |
| 18 | Summarize key results with objectives | Yes | Discussion (p.11) |
| 19 | Discuss limitations, bias, imprecision | Yes | Discussion – Limitations (p.12) |
| 20 | Cautious interpretation | Yes | Discussion (p.12) |
| 21 | Discuss generalisability | Yes | Discussion (p.12) |
| 22 | Funding sources and funder role | Yes | Footer (p.13) |
